# Design and Implementation of a Prosthesis System Controlled by Electromyographic Signals Means, Characterized with Artificial Neural Networks

**DOI:** 10.3390/mi13101681

**Published:** 2022-10-06

**Authors:** David Tinoco-Varela, Jose Amado Ferrer-Varela, Raúl Dalí Cruz-Morales, Erick Axel Padilla-García

**Affiliations:** 1 Engineering Department, Superior Studies Faculty-Cuautitlán, National Autonomous University of Mexico, UNAM, Cuautitlán Izcalli 54714, Mexico; 2 Superior Studies Faculty-Cuautitlán, ITSE, National Autonomous University of Mexico, UNAM, Cuautitlán Izcalli 54714, Mexico; 3 Academy of Robotics Engineering, Polytechnic University of Atlacomulco, Atlacomulco 50465, Mexico

**Keywords:** neural networks, EMG signals, prostheses, embedded system

## Abstract

Around the world many people loss a body member for many reasons, where advances of technology may be useful to help these people to improve the quality of their lives. Then, designing a technologically advanced prosthesis with natural movements is worthy for scientific, commercial, and social reasons. Thus, research of manufacturing, designing, and signal processing may lead up to a low-cost affordable prosthesis. This manuscript presents a low-cost design proposal for an electromyographic electronic system, which is characterized by a neural network based process. Moreover, a hand-type prosthesis is presented and controlled by using the processed electromyographic signals for a required particular use. For this purpose, the user performs several movements by using the healthy-hand to get some electromyographic signals. After that, the obtained signals are processed in a neural network based controller. Once an usable behavior is obtained, an exact replica of controlled motions are adapted for the other hand by using the designed prosthesis. The characterization process of bioelectrical signals was performed by training twenty characteristics obtained from the original raw signal in contrast with other papers in which seven characteristics have been tested on average. The proposed model reached a 95.2% computer test accuracy and 93% accuracy in a real environment experiment. The platform was tested via online and offline, where the best response was obtained in the online execution time.

## 1. Introduction

In 2017, around 57.7 million people in the world suffered the amputation of a limb due to traumatic cases [1]. This means that affected people see the quality of their lives diminished, being of great importance to design prostheses that may be set to each particular situation. Moreover, the design of prostheses which can be controlled and can react in a “natural” way, regarding the required complex motions of mechanisms, is of paramount interest.

For this purpose, the use of bioelectrical electromyographic (EMG) signals is proposed as element of control. EMG signals are generated by every muscular contraction. Then, the user could control the prosthesis in the same way that would react if had the body member.

From above, development of bio mechatronic prosthesis with EMG signals is presented in this manuscript, being a way to get each movement and particular bioelectrical representation that can be characterized to obtain signals that can be used as commands.

To treat EMG signals, an adequate amplitude range was obtained by amplifying them after having acquired values directly through an electrode. Thus, the signal amplitude varies from μV to a low range of mV (lower than 10 mV), and according to Trimedica [2], the usable energy of a signal is bounded in the frequency range of 0–500 Hz with dominant energy in the range of 50–150 Hz, both ranges above the electrical noise level. To obtain a filtered sensory system, a converter was proposed for the raw signals (without processing) to adequate them into suitable signals for characterization and classification, i.e., subject the acquired signal to a cleaning process.

It is worth to mention that the main drawback of a system that characterizes EMG signals is the sensitivity of the usable signal profiles which depend on different factors, such as the muscle contraction intensity, the electrodes position, and on the skin type and condition, additionally, measurements are affected by the collection data system conditions, the electrode quality, sensor design, environment noise, etc. Moreover, instrumentation, software, sensors, and specific devices to face this set of factors would make it hard to develop a low-cost experimental platform. To face with this set of factors, the system can be characterized by obtaining as much as possible information of signal samples that belongs to the training dataset of the artificial neural network (ANN).

In literature, EMG signals have been used in several experiments, for instance, EMG signals have been used to detect emotion’s reaction signals as in [2], to create musical sounds as in [3] and to develop smart clothes [4]. Also, EMG signals have been used to get control parameters of robotic arms [5], to operate MP3 players [6], and mobile phones [7], to characterize gestures [8], and for applications in human-machine interfaces [9].

Gesture or manual movement classification is one of the goals of this work since it seeks to develop a system that adequately classifies the EMG signals of the user, and to use that classification to control a hand prosthesis. Some different types of classification that can be reviewed from the literature, and some of those works related to this proposal are the following. The most common are by using support vector machines [10] random forest [11], k-nearest neighbors (k-NN) [12], wavelet transform [13], decision trees [14], fuzzy logic [15,16] and artificial neural networks [8], among others.

Artificial neural networks have become very popular for characterization and classification, and they have been used in different projects to classify hand movements to design prostheses, where a fluid drives the “natural” movement of the human body [17,18,19]. Regarding EMG signal characterization, raw signals and extraction of features can be used as input elements [20], where pattern classification success depends on the features selection. Vectors composed by characteristics of the original EMG signal have been used as inputs to train neural networks, where the main used values are the mean absolute value (MAV), variance (VAR), standard deviation (SD), zero crossing (ZC), and the root mean square (RMS). However, there are some other characteristics of the EMG signals to be used, such as waveform length (WL), mean absolute value slope (MAVS), mean frequency (MNF), median frequency (MDF), slope sign change (SSC), root mean square (RMS), wavelet packet transform (WPT) coefficients, auto regression (AR) coefficients, Willson amplitude (WA), fast Fourier transform (FFT) coefficients, cepstrum coefficients (CC), and short-time Fourier transform (STFT) coefficients. In this manuscript, 20 characteristics of the EMG signals are extracted and used as inputs for an ANN, to enrich the characterization and classification process, unlike other works that typically use just 7 characteristics.

It is worth to mention that all the design, filtering, and sensor characterization have been carried out by using a low-cost system, inferring that no expensive devices are used for its implementation. Then, this proposal leads to an affordable solution, compared to other proposals in literature, where the production costs are more expensive. Moreover, our system reached a 95.2% of precision in the computer evaluation and 93% in the real test. Additionally, it was tested for online and offline experiments, where the best system response was obtained by online execution time.

This manuscript is organized as follows, in Section 2, the preliminaries of the project realization are presented; in Section 3, the proposed design of the system is stated, where the characteristics extraction of the raw EMG signal, the input vector of used neural network, and the signal process development are described. In Section 4, experiments and data acquisition are shown, analyzing the obtained results by showing a comparison with other similar proposals. Finally, in Section 5 the conclusions to analyze the improvements obtained and future work are stated.

## 2. Preliminaries

This section presents the preliminaries for better understanding of the proposed prosthesis. Focusing on artificial neural networks and electronic filters.

### 2.1. Filters

**Figure 1:**
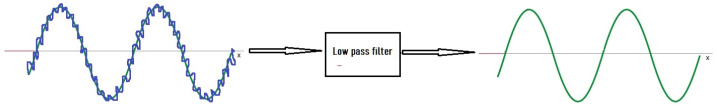
Using a low-pass filter to remove noise from a sinusoidal signal.

Electronic filters allows passing or suppressing a certain set of frequencies, such as low-pass (signals with high frequencies are suppressed), high-pass (signals with low frequencies are suppressed) and band-pass (allows the passage of a set of frequencies, delimited by a lower and an upper limit). For instance, in Figure 1 a low-pass filter was used to decrease the presented noise of the signal, allowing only the one belonging to the required signal.

Depending on the type of components used, there are passive and active filters. The passive ones only contain resistive, capacitive, and inductive elements, while the active ones contain active components, that are typically operated and tuned by using operational amplifiers. Regarding active filters, three of the most used active filters are: Butterworth, Bessel, and Chebyshev. If an ideal low-pass filter existed, all the active filters would eliminate signals above the cutoff frequency, and perfectly pass signals below the cutoff frequency. However, in a real situation, many trade-offs should be considered to get the optimum performance for a given application.

In this work, the Butterworth filter is used to obtain the required signals of the user body, since they are easy to handle at many real time applications [21,22]. Then, these obtained signals are used on the artificial neural network.

### 2.2. Artificial Neural Networks

**Figure 2:**
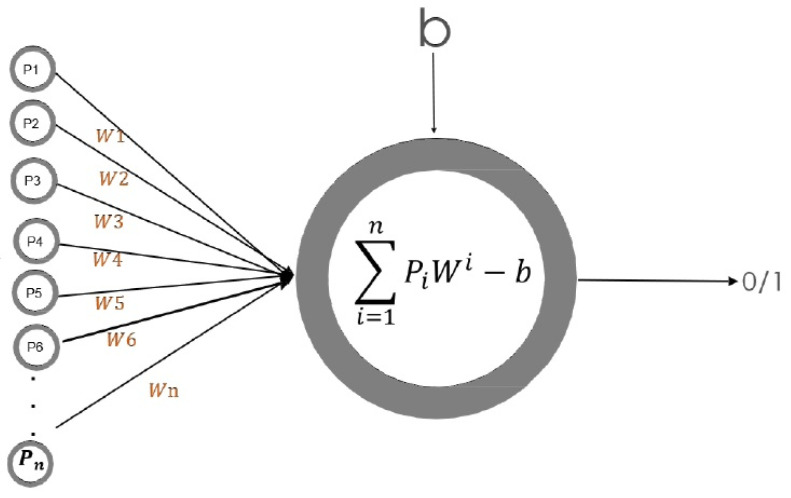
Perceptron or basic artificial neuron.

The simplest model of a biological neuron is the well-known perceptron, introduced by Frank Rosenblatt, which emulates a neuron behavior by reproducing its main characteristics. In this model, the information reception through different inputs $P_{1},P_{2},\cdots ,P_{n}$, as they were dendrites to stimulates the neuron nucleus, and the final value depends on an active response (1) or inactive response (0) of the neuron. Thus, the mathematical representation of the neuron activation function can be defined as

$$\begin{array}{c} S=\Sigma_{i=1}^{n}P_{i}W_{i}-b\\ Output=0\;\;if\;\;S\leq 0\\ 1\;\;if\;\;S\geq 1 \end{array}$$

where $W_{1},\cdots ,W_{n}$ are the synaptic weights, and *b* is the bias of the neuron. A schematic representation of perceptron can be seen in Figure 2.

In this work, a supervised learning was implemented for the training process of the neuron, i.e., the output values for a set of input data are known by the designer. Then, if the expected output *z* is different from the obtained output *y*, an error e=z−y≠0 is obtained and the synaptic weights of the neuron are adjusted as result of this error. Such that, $W_{i}^{{}^{\prime }}=W_{i}+\gamma eX_{i}$ where γ is the learning factor, and $X_{i}$ are the inputs.

This adjustment is performed while e≠0. When e=0 in all cases, a trained neuron is obtained. From this neuron, that is the minimum unit, more complex configurations can be built by interconnecting them, giving way to artificial neural network architectures.

**Figure 3:**
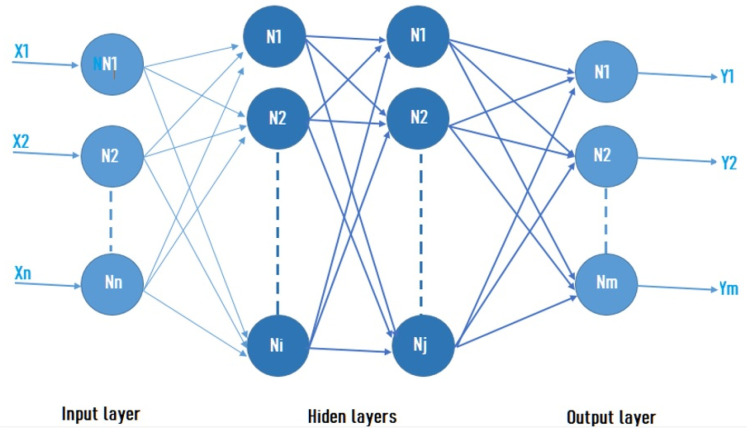
Representation of an ANN with n inputs and m outputs.

An ANN is a computational scheme that emulates the learning process of a biological brain. Also, the ANN are connectionist systems, i.e., interconnection of different independent neurons is performed, until a network of neurons communicated by means of synaptic weights is arrange, a neural network general scheme can be seen in Figure 3, which basically consists of the input layer, the output layer, and the hidden layers. The neurons number in the input layer is conditioned by the number of elements in the input vector with which was trained. The number of neurons in the output layer corresponds to the classes or classification sets number that the problem contains. Finally, the neurons number in the hidden layers will depend on the nature and complexity of the addressed problem.

Before an ANN can classify a behavior or structure, it must be trained, which means that a user must told it what to learn. To carry out this training, it is necessary to enter many phenomena or problem known cases to be learned, the network will adjust itself in such way that it will learn that for a set of inputs, there is a certain output. The cases with which a network is trained are stored in a dataset, and the ANN will obtain all the information from such dataset.

These schemes have been used mainly in classification problems [23], object detection [24], image processing [25], and to estimate sustainable transport demand [26], controllers [27,28], melanoma detection [29], among others.

## 3. Materials and Methods

This section presents the entire design and development process of the proposed system, from the electronic to the artificial learning part and its implementation. The neural network that controls the prosthesis needs to be fed by a database to work and be able to learn to replicate the desired movements. In this case, the signals database is captured from the user who performs different muscle contractions movements. For this reason, it is necessary to design an electromyographic signal sensor that obtain and adapt such signals to be processed and stored, in order to be used for training the neural network. The design of this sensor is presented.

### 3.1. Electromyographic Sensor Design

To design an electronic device capable of sensing small electromagnetic variations, the obtained EMG signals were amplified, filtered, and conditioned to be used by an Arduino board.

To replicate the movements for a hand, different types of sensing methods can be used for this purpose, such as cyber gloves [30], cameras [31], and electromyography [32]. The electromyography was the used method in this paper. Despite the available EMG sensors on the market, such as Myoware and Oymotion, in this proposal the own EMG sensor is made to obtain a raw signal for the Arduino development board. To design this sensor, five stages are considered to condition the signal: pre-amplification, signal filtering (low-pass and high-pass filters), final amplification, and offset. Each stage in the signal cleaning process is described as follows.

### 3.2. Signals Conditioning

#### 3.2.1. Pre-Amplification

This stage is the first contact with the signal and the subsequent processes. Thus, instrumentation amplifiers are used, and their working conditions are design by using ideal amplifiers conditions, as infinity input impedance, zero impedance output, high ratio rejection, and highly gain voltage, all this to prevent added noise to the amplified signal. An amplifier of this type can be purchased commercially, however, an own amplification system is designed to adapt it to the needs of the project, this is done by using LM833 operational amplifiers.

The general amplification circuit, based on the EMG sensor, is divided in two amplification stages, due to, if all the amplification is carried out in a single stage, saturation would be generated in the output, and consequently an EMG signal loss.

**Figure 4:**
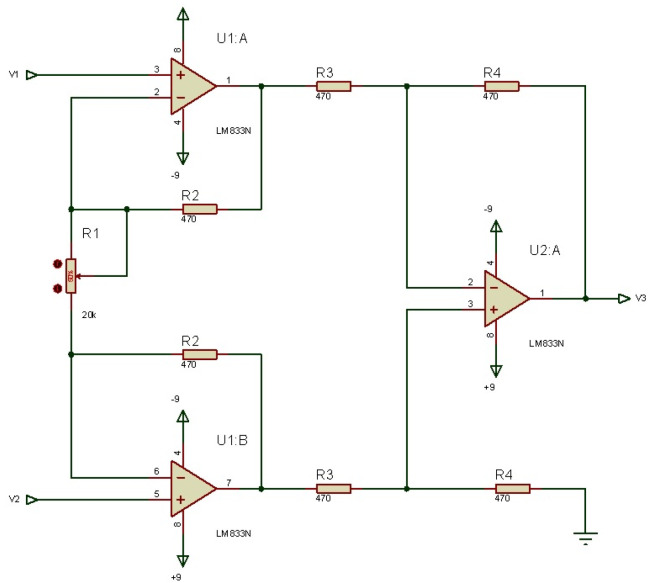
Schematic of the amplifier circuit with LM883.

Figure 4 shows the amplification circuit designed with LM833, which serves as a signal pre-amplifier.

Where *V*3 is the output signal given by,

(1) $$V3=\left(V2-V1\right)\left(\frac{R4}{R3}\right)\left(2\left(\frac{R2}{R1}\right)+1\right)$$

where V2 and V1 are the circuit’s input voltages, $R_{1},R_{2},R_{3},R_{4}$ are resistances for amplification setting.

From Equation (1), it is possible to notice that the gain depends on $R_{2}$, but the variation depends on $R_{1}$, if $R_{3}=R_{4}$ is considered.

#### 3.2.2. Third Order Low-Pass Butterworth Filter

**Figure 5:**
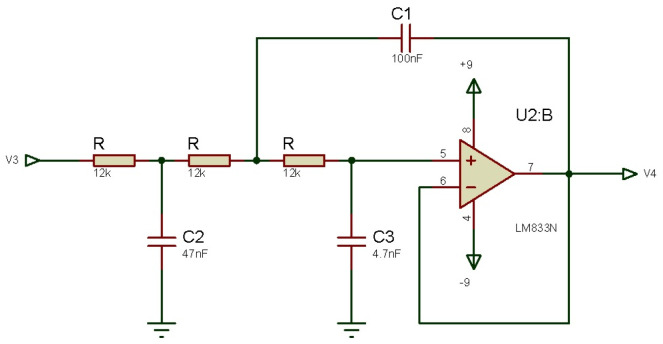
Schematic diagram of a 3rd order low-pass Butterworth filter.

A raw signal is susceptible to noise and contamination, so it should be conditioned before using it, for that reason a pre-amplification stage is proposed where a Butterworth active filter is used to filtering and discarding frequencies and values that do not give a representative value on the input signal. Later, the following must be considered: higher order filters, use a higher number of operational amplifiers, resistors, and capacitors. Considering this, it is decided to design a third order filter, which needs less components, and has an adequate cutoff frequency response. A cutoff frequency of 500 Hz is established to eliminate noise caused by electronic equipment and electronic components of the sensor itself. Figure 5 shows the designed filter. A simulation of this filter is performed by using R=12 kΩ, $C_{2}=47$ μF, $C_{3}=4.7$ μF, and a LM833N amplifier where is obtained that the real cutoff frequency is at 471 Hz, since the EMG frequency interest range is between 0 to 500 Hz and the greatest amount of information is between 50 Hz and 150 Hz, this cutoff frequency is acceptable. This filter has a 61 dB drop per decade.

#### 3.2.3. Third Order High Pass Filter

**Figure 6:**
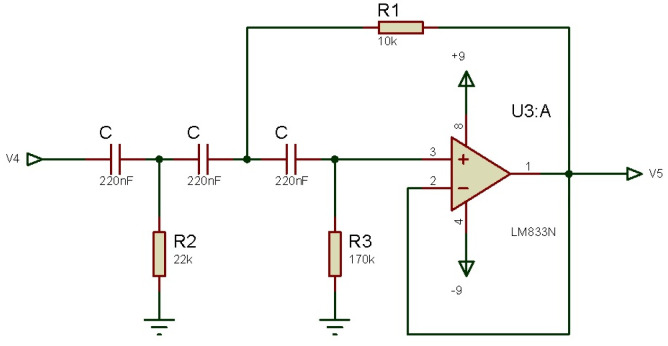
3rd order high-pass butterworth filter.

A high pass filter should be designed for better signal matching and handling. Then, a cutoff frequency of 20 Hz is established, this cutoff frequency is selected to eliminate the noise caused by the movement of the cable that connects the electrode and the skin. The electrical signals from both noise sources have most of their energy in the frequency range from 0 to 20 Hz. The designed circuit is shown in Figure 6. Simulation of this filter is carried out to obtain that the real cutoff frequency is 22.7 Hz, the filter has a drop of −60.3 dB per decade.

#### 3.2.4. Non-Inverting Amplifier

**Figure 7:**
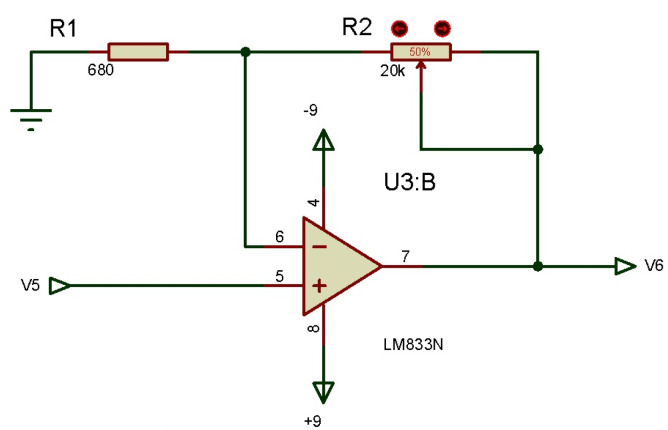
Non-inverting amplifier schematic.

In the second amplification stage, a non-inverting amplifier is made, which is designed to deliver a minimum gain of 1 and a maximum gain of 30 according to the $R_{2}$ potentiometer value of 20 KΩ, the amplifier design is shown in Figure 7.

#### 3.2.5. Offset (Non-Inverting Summing Amplifier)

**Figure 8:**
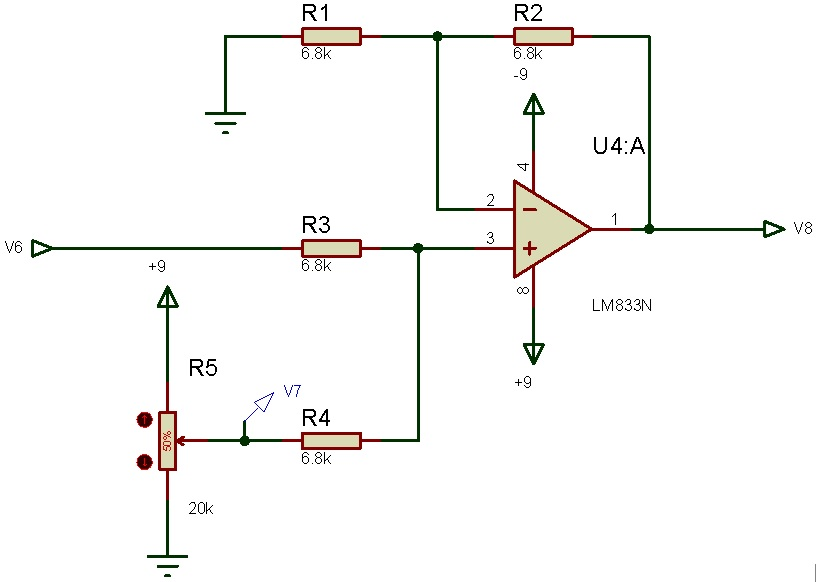
Designed offset circuit.

Once the signals are amplified and filtered, an EMG signal is available, which consists of positive and negative voltages, but is not compatible with the chosen microcontroller device, Arduino. To adapt this signal and avoid microcontroller damage, one last conditioning signal stage is necessary, consisting of a non-inverting summing amplifier. An offset circuit that equalize all the resistances, raises the potentiometer’s voltage, this allows placing the signal at 2.5 V as a new reference, obtaining an EMG signal ready to be processed by the Arduino, the designed offset circuit from 0 V to 9 V is shown in Figure 8.

### 3.3. Neural Network Design

Considering that the amplified signals are obtained and adapted, they are stored within a dataset and used for training the neural network, however, for a better neural network learning and classification, is necessary to extract certain signal characteristics so that the ANN training be adequate and functional. Due to this, using different techniques and algorithms, 20 main characteristics are extracted from each user signals obtained. When these characteristics are obtained, a representative vector is created, and stored in a dataset training and therefore feeds the neural network input.

#### Extraction of Features

The extraction of characteristics is recommended since they are chosen according to the needs of training and operation of the neural network, for this reason the characteristics extracted from the raw EMG signals and the way to obtain them are described. The 20 characteristics extracted from the raw EMG signal to create the input vectors, in order to train the neural network. Table 1 shows the selected characteristics to be extracted, then a brief explanation of the way in which each of them are calculated is shown in this section.

**Mean absolute value (MAV):** It is the sum of each segment ${\bar{x}}_{i}$ of *N* samples of a signal. Where $x_{k}$ is the *k*-th sample in segment *i*, and *I* is the total number of segments.

$${\bar{x}}_{i}=\frac{1}{N}\Sigma_{k=1}^{N}\left|x_{k}\right|,\;\;i=1,2,\cdots ,I$$

**Zero crossing (ZC):** It is the number of times the waveform crosses zero. To obtain the result, a comparison is made between two consecutive sections of the signal $x_{k}$ y $x_{\left(k+1\right)}$, such that:

$$\begin{array}{c} x_{k}>0\;\;and\;\;x_{\left(k+1\right)}<0\\ x_{k}<0\;\;and\;\;x_{\left(k+1\right)}>0 \end{array}$$

A work related to this concept is presented in [33], which is focusing on EMG signals.

**Slope sign change (SSC):** Similar to ZC, but now measuring sign changes between signal elements.

$$\begin{array}{c} x_{k}>x_{\left(k-1\right)}\;\;and\;\;x_{k}>x_{\left(k+1\right)}\\ x_{k}<x_{\left(k-1\right)}\;\;and\;\;x_{k}<x_{\left(k+1\right)} \end{array}$$

**Waveform length (WL):** It is the sum of the differences between values of consecutive samples of the EMG signal, up to the last sample. It is obtained as follows.

$$\begin{array}{c} wl=\Sigma_{k=1}^{N}\left|\Delta x_{k}\right| \end{array}$$

where $x_{k}=x_{k}-x_{\left(k-1\right)}$.

**P-order auto regressive model AR(P):** It is used for a random process representation of a model in which the main variable depends on its past values and it is possible to obtain the coefficients of the auto-correlation function $a_{i}$, P gives the order, and e(k) is the element error.

$$\begin{array}{c} x\left(k\right)=\Sigma_{i=1}^{P}a_{i}x\left(k-i\right)+e\left(k\right) \end{array}$$

**Fast Fourier Transform (FFT):** Fourier transform allows a signal to be broken down into its individual components, in such way, it is possible to obtain information that would otherwise be hidden itself within the signal. It is defined as:

$$\begin{array}{c} X_{k}=\Sigma_{n=0}^{N-1}x_{n}e^{-\frac{2\phi i}{N}kn},\;\;\;\;k=0,\cdots ,N-1 \end{array}$$

where $x_{n}$ is the sampled signal for specific discrete time. A fixed period time sampling was taken to obtain the estimate of the power spectral density, and thus have the characteristics of energy and average power.

**Short time Fourier transform (STFT):** It separates the original signal into sections, each of these sections is analyzed by means of the Fourier transform, and each section characteristics are extracted independently. This analysis can be obtained as follows:

$$\begin{array}{c} STFTx(t)(\tau ,\omega )\equiv X\left(\tau ,\omega \right)=\int_{-\infty }^{\infty }x\left(t\right)w\left(t-\tau \right)e^{-i\omega t}dt \end{array}$$

where w(t) is the window function.

Spectrogram consists of acquiring a certain number of samples through a temporary window, with a specific size. The frequency content is determined for these samples, and they are represented in a three-dimensional graph. Performing this same operation on the total length of the signal. This process gives us frequency information and the variation of energy. For this study, the short Fourier transform of 3-time windows of the EMG signal is obtained, using a standard rectangular window, from each, two characteristics are extracted, the energy and the average power, elements that compose the input vector of the ANN to train the system.

**Discrete Wavelet Transform DWT:** Wavelet continuous transform (CWT), express a continuous x(t) signal as an expansion of coefficients proportional to the product between the signal x(t) and the scaled and displaced version of the mother wavelet $\psi_{\left(a,b\right)}$,

$$\begin{array}{c} CWT\left(a,b\right)=\int_{-\infty }^{\infty }x\left(t\right)\psi_{\left(a,b\right)}\left(t\right)dt\;\;\;\;a,b\epsilon \;R\\ \psi_{(}a,b)=\frac{1}{\sqrt{\left|a\right|}}\psi \left(\frac{\left(t-b\right)}{a}\right) \end{array}$$

where *a* is the scale parameter to compress or expand and *b* is the translation parameter that moves the Wavelet. Discrete wavelet transform (DWT) is obtained by discretizing the CWT. The values $a=2^{-j}$ and $b=k2^{-j}$ are usually used to discretize for parameters *a* and *b*, hence the mother Wavelet takes the form:

(2) $$\begin{array}{c} \psi_{\left(j,k\right)}\left(t\right)=2^{\frac{j}{2}}\psi \left(2^{j}t-k\right) \end{array}$$

Thus, the Wavelet ψ(t) with a scale function ϕ(t), is associated, which allows us to approximate any function x(t) with these functions, by means of the expression:

(3) $$\begin{array}{c} x\left(t\right)=\Sigma_{k}\Sigma_{j}c_{\left(j,k\right)}\phi \left(t\right)+\Sigma_{k}\Sigma_{j}d_{\left(j,k\right)}\psi \left(t\right)\;\;\;\;j,k\epsilon Z \end{array}$$

where $c_{\left(j,k\right)}$ are the scale or approximation coefficients, and $d_{\left(j,k\right)}$ represents the Wavelet or detail coefficients of the original signal x(t), with respect to the scale function ϕ(t) and the Wavelet function ψ(t).

The behavior of the DWT is like a “filter banks”. Such filters correspond to a low-pass and a high-pass filter, when the original signal passing through them, the output coefficients $c_{\left(j,k\right)}$ and $d_{\left(j,k\right)}$ respectively, are obtained. A decomposition of the signal can be obtained at different levels by passing the scale coefficients obtained from the previous filtering by an identical pair of filters, thus obtaining the coefficients of the next level.

The Wavelet selected for this article is *Daubechies D5*, which will provide us with five approximation coefficients and five detail coefficients, for each of the 5 approximation coefficients the variance calculation has been performed.

### 3.4. Experimental Platform

**Figure 9:**
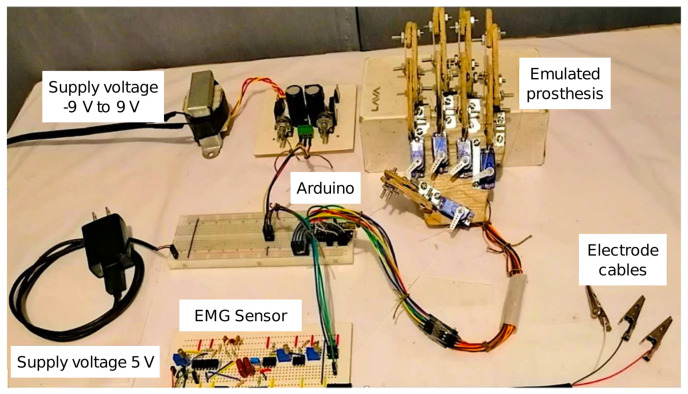
Experimental platform for a bionic−hand prosthesis.

The experimental platform is shown in Figure 9, and it consists of an Arduino Nano board, this card has an ATmega328 microcontroller of AVR architecture. The analog to digital converter has a resolution of 10 bits, with a conversion range voltage of 0 V to 5 V. Pins 5, 6, 9, 10, and 11 are used for servomotor control, and analog pin 2 for EMG signal acquisition.

To obtain the EMG signals, low-cost self-adhesive silicone gel electrodes are used, which are reusable, 10 of these worth approximately 7.5 USD. A complete cleaning must be carried out in the area to be placed to keep the skin clean each time a user wears it.

**Figure 10:**
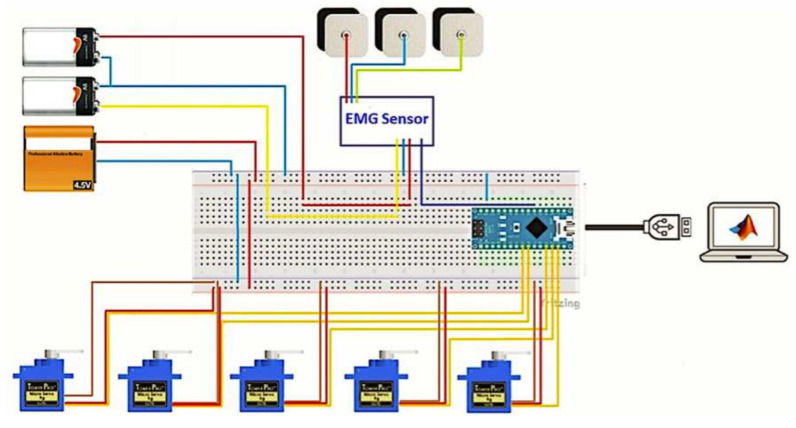
Electrical diagram connection of experimental platform for human-hand emulation.

In Figure 10 the electronic circuit schematic built for the control of the hand is presented. Additionally, a complete amplification circuit for sending filtered and conditioned signals to the microprocessor is performed by using a perforated phenolic prototype board.

## 4. Results

This section shows the implementation and tests carried out with the EMG signals sensor. To carry out the tests, Simulink/MATLAB software version 2020, Mathworks, (MATLAB Central, Mexico) was used together with the *Toolbox Simulink Support Package* for Arduino Hardware, with this it is possible to carry out oscilloscope simulations and correctly adjust the signals from electrodes. This is done by adjusting the three potentiometers with the resistive values described in the design of the high-pass and low-pass filters.

### 4.1. Implementation and Testing of EMG Sensor with MATLAB

Users must place the electrodes on the arm to obtain the system signals and send them to the computer that are displayed through an oscilloscope made in Simulink/MATLAB.

**Figure 11:**
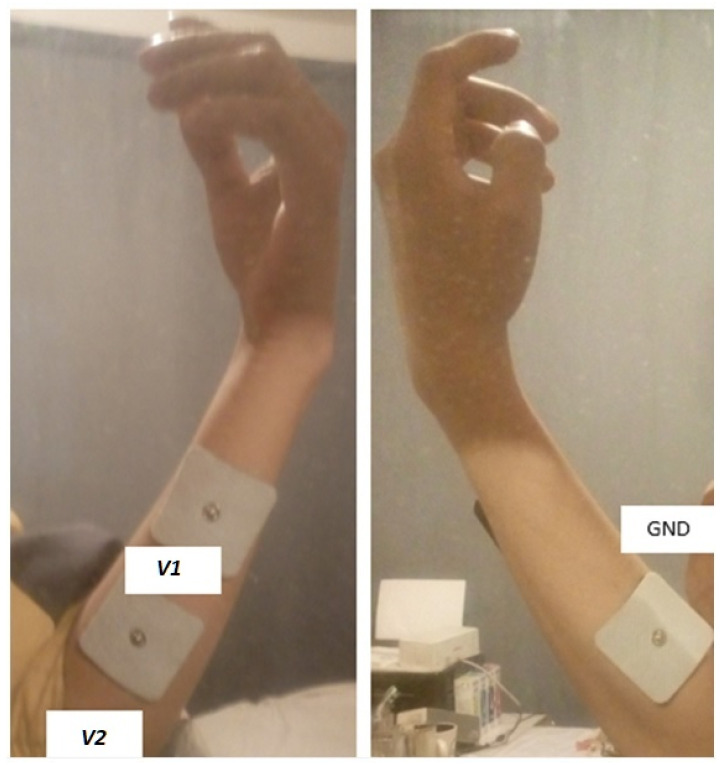
Location of the electrodes on the arm.

Figure 11 shows the used position to extract the EMG signals, where the *V*1 and *V*2 electrodes must be placed in the muscle in which the EMG signals are acquired, and the GND electrode in a nearby area. It is important to emphasize that it must be an area where the muscle is not working. The proper location for the electrodes is between the innervation zone (or motor unit) and the tendinous insertion, or belly of the muscle [34].

**Figure 12:**
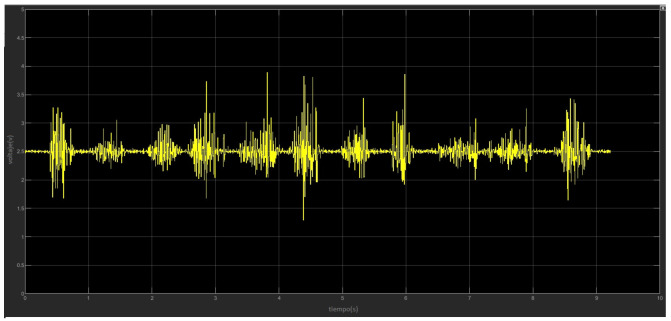
EMG signal acquired by the developed sensor and displayed in the virtual oscilloscope designed in Simulink. The signal is traced in real time.

Once the EMG signals are obtained, they are displayed on the oscilloscope designed in Simulink, adjustments are made to adapt the values and the signal can be acquired properly. The final values are as follows, for a gain of 31 in the preamp stage, potentiometer is adjusted to 27.64 Ω and for a gain of 21 in the final amplification, potentiometer is adjusted to 13.6 KΩ, giving a total gain of 735. It is also important to notice that the final applied offset is 1.5 V with a potentiometer resistance of 3.3 KΩ. Then, the gains are tuned, tests are carried out that consist of moving the fingers of the hand with small force and the acquired signals can be observed in MATLAB oscilloscope as shown in Figure 12.

### 4.2. Data Acquisition

Once the signal is acquired satisfactorily, the integrated microcontroller development environment (IDE) is used to create a program that process the muscles signals sent by the electrodes. The acquired signal is discretized by the acquisition system and is important to guarantee that the signals characteristics can be reconstructed, for that reason, the Nyquist theorem is used, this theorem indicates that the sampling frequency must be at least twice than the frequency of the signal that will be reconstructed. The frequency limits that the designed EMG sensor circuit admits go from 22.7 Hz to 472 Hz, where

$$\begin{array}{c} f_{sample}\geq 2f_{signal}=2\times 472\;\mathrm{Hz}=944\;\mathrm{Hz} \end{array}$$

The sampling velocity of created program is 1 kHz, this signal is acquired each time it is requested by MATLAB, the number of samples sent is 60,000 in 60 s, these are sent through the serial port. Each sample generates 2 bytes, so a total of 120,000 bytes are sent over 60 s.

**Figure 13:**
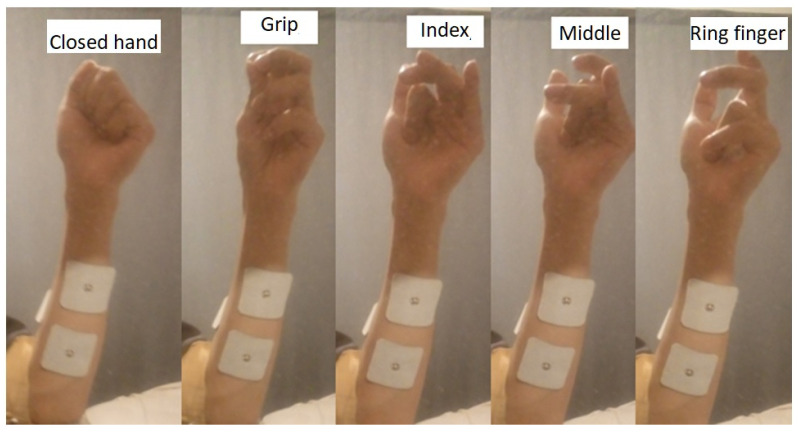
Positions of the hand and fingers that are characterized.

A Simulink/MATLAB interface was created, which is responsible for receiving the sampled signal by the Arduino board, this interface stores the information in matrix structures. Each hand movement generates a particular signal, these are stored in a labeled matrix to identify which movement was generated, these are: closed hand, grip, index, middle and ring finger movement. These movements are shown in Figure 13.

**Figure 14:**
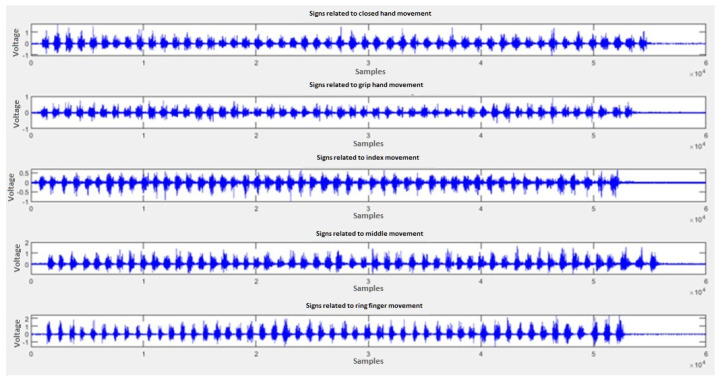
Samples sequences related to each hand movement to be characterized, obtained from the sensor and plotted by Simulink.

We generated a database or “dataset” with the signals of each of the described movements, 50 samples of each movement are collected, with 500 sampling points each. Once obtained, sequences of the same movement are generated and saved as a single vector. Figure 14 shows the obtained sequences.

**Figure 15:**
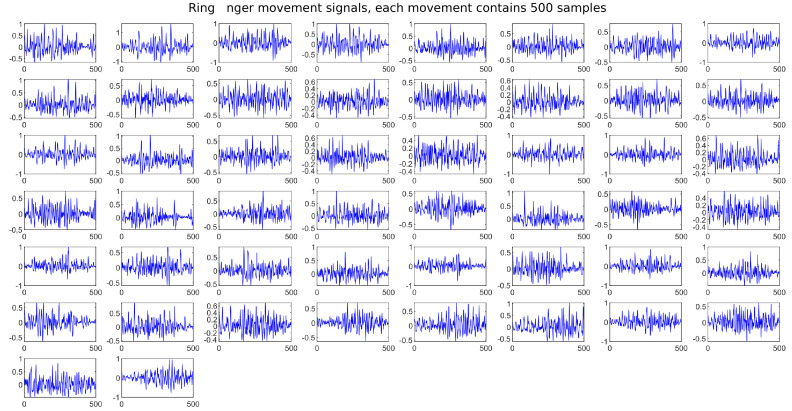
Signals with the windowing process obtained from each movement to be characterized.

A windowing process is applied to each of these signals (Figure 15), which consists of delimiting only the area of interest and cutting the linear part that does not have information of the muscular effort to be analyzed. In this way, all 500 samples are saved, and the storage is optimized by reducing each signal to only the part of interest. Such signals are stored in arrays for later analysis within a neural network.

Finally, dataset is generated with each structured vector from the extracted characteristics of each movement signal, previously defined and the input array for the neural network is created. In total, 250 signals are obtained, which are divided into 250 vectors of 20 characteristics each, therefore, there is a 20 × 250 matrix.

### 4.3. Training of the Neural Network

This section describes the neural network process training with the dataset obtained and described in Section 4.2.

**Figure 16:**
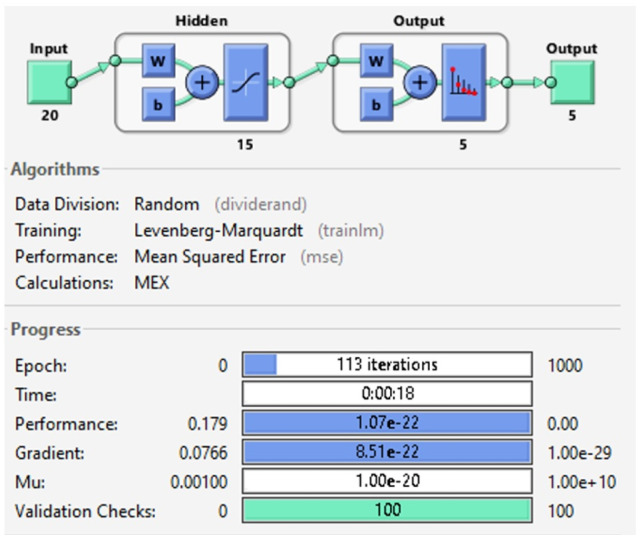
Architecture and training characteristics of the ANN. Note: In this figure 8e+3 represents $8\times 10^{3}$ in scientific notation.

For the hand movements characterization, a supervised neural network for pattern recognition is created, with a trainlim training function. The network has a 20 neurons input layer, automatically adjusted by the algorithm according to the dataset, there is a 15 neurons hidden layer defined in the program, with a sigmoid activation function and a 5 neurons output layer with a gradient descent function. In Figure 16, the network architecture is shown, as well as its training parameters.

**Figure 17:**
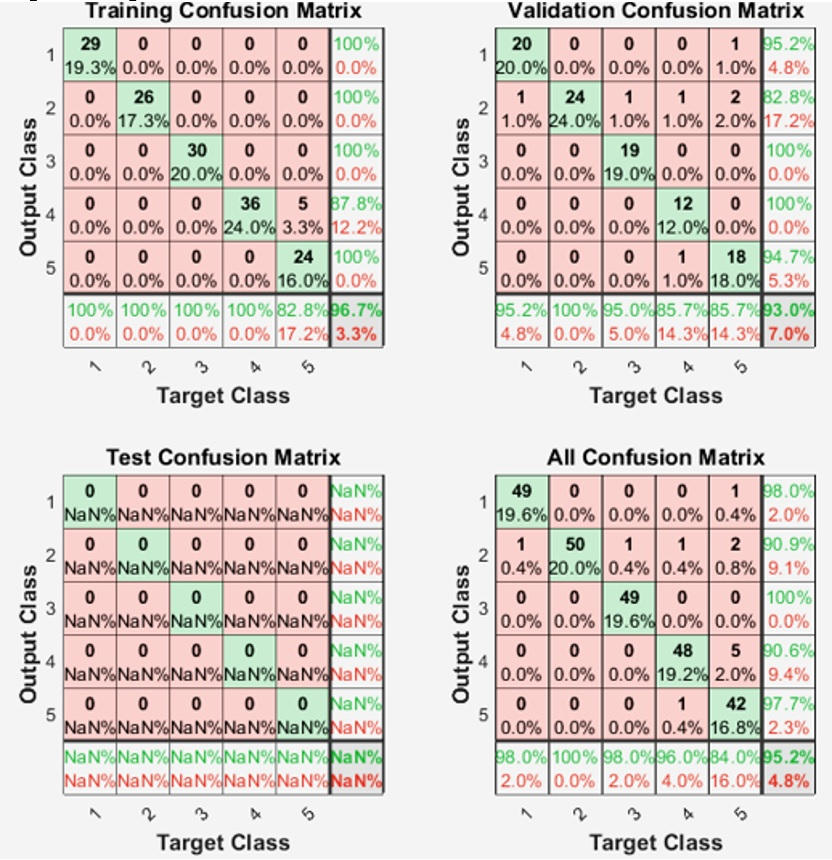
Confusion matrix related to the training and validation of the characterized EMG signals.

The original dataset was divided in two subsets: 60% for training data and 40% for validation, discarding a test subset. From these sets, the generated confusion matrix is observed in Figure 17. It can be seen that in the characterization of these 5 movements, an adequate response was obtained, which achieves a success rate of 95.2%.

Notice that before select the training function (TF) mentioned previously, it was necessary to compare and choose from different TF. It is difficult to obtain the best neural network architecture to solve a problem, however, tools like MATLAB has efficient training function which can be used to determine the best suited to our problem. In this case, five TF were analyzed in order to determine which one would be used on the prosthesis. The different TF were implemented in a feedforward neural network.

To make the selection process of the best TF, a database with the hand movements signals has been used, the dataset has been divided into 60% training, 20% validation and 20% testing. The executed TF’s and the data obtained from each are shown in Table 2.

Table 2 shown that the Levenberg-Marquardt algorithm corresponding to the trainlm TF, presents the best precision results, being the algorithm used in this approach.

**Figure 18:**
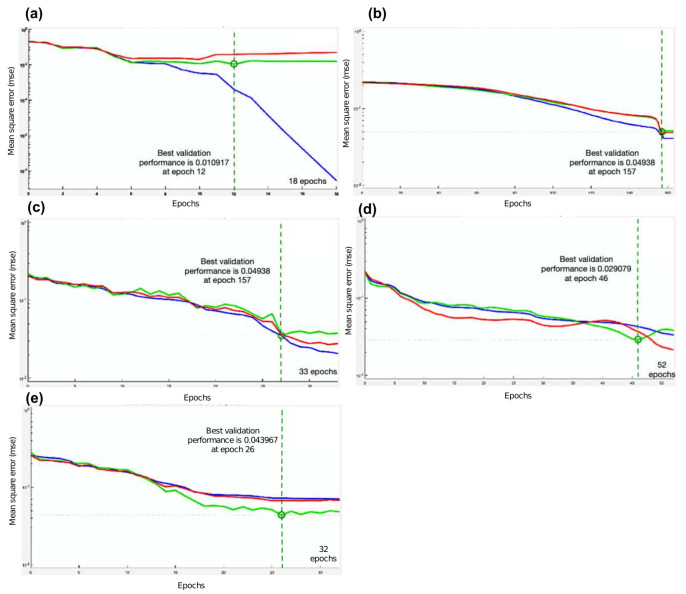
Evaluated TF’s performance where blue line is the training, green line the validation, red line the test, and dot line the best validation performance for 5 case studies: (**a**) Trainlm, (**b**) Trainbfg, (**c**) Traingcp, (**d**) Trainoss and (**e**) Traingdx.

**Figure 19:**
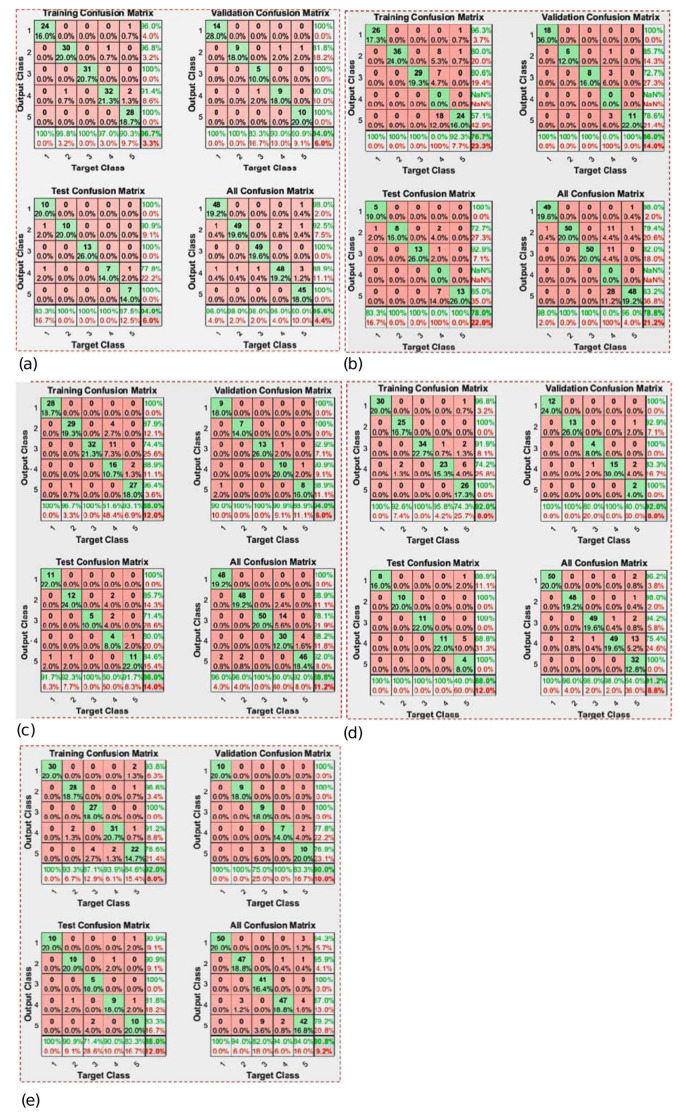
Evaluated TF’s confusion matrix. (**a**) Trainlm, (**b**) Trainbfg, (**c**) Traingcp, (**d**) Trainoss and (**e**) Traingdx.

Figure 18 shows the performance metrics for each of the TFs, and Figure 19 shows the confusion matrices for each of these TFs.

### 4.4. Emulation of a Human Hand

**Figure 20:**
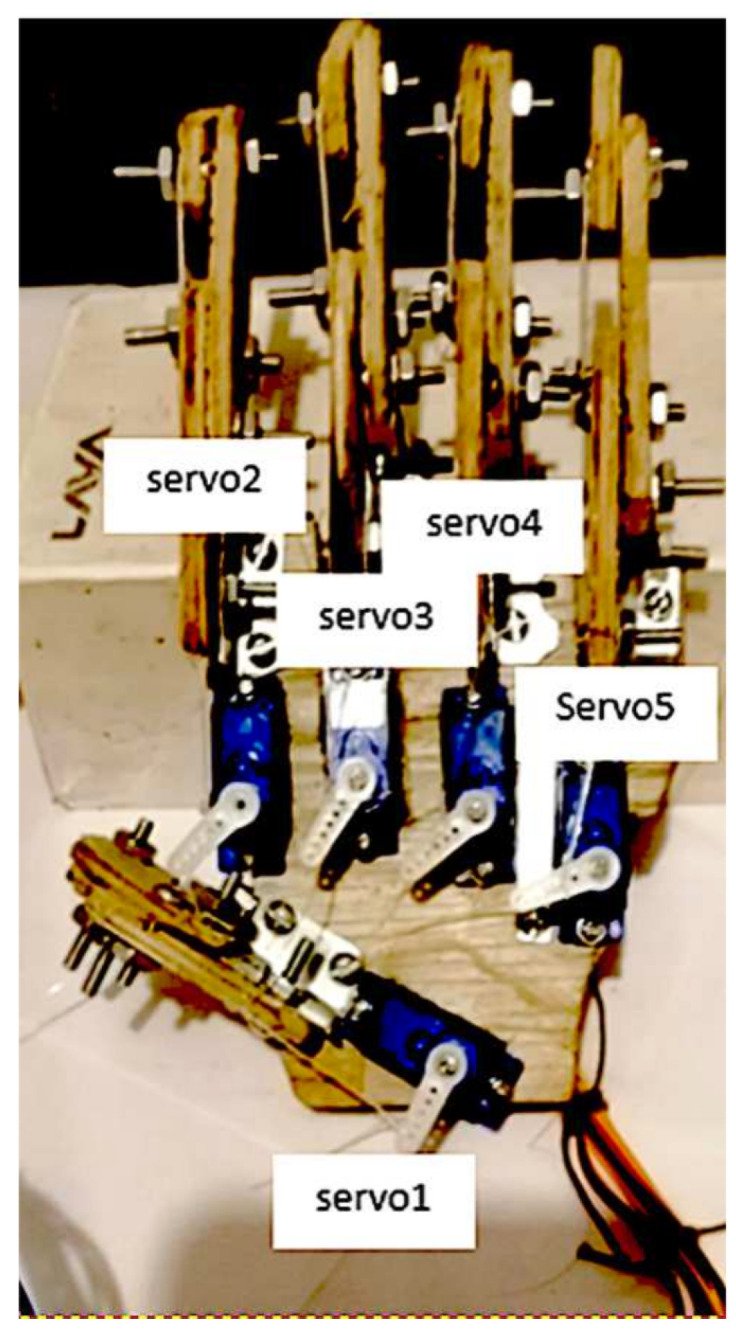
Bionic-hand prosthesis which emulates human-hand movements.

With the obtained signals and the hand movements characterized by the previously described neural network, a human hand prosthesis was created to verify the operation of the ANN that emulates the hand movements. In order to physically check the movements generated by the neural network, a prosthesis is developed that consists of 5 servomotors, 5 fingers with three phalanges each, which mimic the fingers and hand movements. The neural network sends the necessary signals to the servomotors to replicate the indicated movements, this developed prosthesis is shown in Figure 20.

Real environment operational test is carried out by performing 90 random movements, taken from the previously described in Section 4.2. Where 84 movements were correctly replicated and 6 were incorrectly performed, obtaining a 93.3% of assertiveness and 6.6% of error. This is according with the efficiency percentages obtained in the training and validation.

The EMG signal characteristics extracting, and neural network training methods are compared with others to analyze and demonstrate that the used method has an adequate response and is at least as effective as the most efficient known methods. This comparison is shown in Table 3.

This proposal shown similar efficiency to the others, despite having more features per input vector, the efficiency has not been significantly increased. This is interesting, since it could be deduce that there is a maximum number of characteristics that significantly influence the way in which neural networks learn the behavior of EMG signals, a problem that will be investigated in a future work. Notice that the acquisition and experimentation system is a low-cost designed, even so compared to other prototypes and authors that used more expensive EMG sensors our present better response. This fact can lead to the conclusion, that hardware may not have such an influence on the EMG signals characterization success and that the efficiency rate depends only on the neural network training and learning.

### 4.5. Online Execution

Online system testing is performed by using the MATLAB Online tool in which the same offline code is used and the same data is loaded in order to verify the behavior of our system in an online environment. Which will be used as a node of the Internet of things in a future work.

To obtain the system’s efficiency in the online environment, about 70 random tests are carried out, where a better behavior in temporal response is obtained. Table 4 shows online and offline tests, the time showed on it, is a mean value.

It can be seen in Table 4 that the online execution time is better than offline execution, in this case, the algorithm was executed in a computer with 6 GB DDR RAM memory and an AMD A8-6410 processor running at 2.4 GHz, which can be an important factor to obtain such results. Notice that the online execution process controls the designed prosthesis in the same way as the offline execution, which is an important fact related to our system.

## 5. Conclusions

This manuscript presents an entire EMG signal acquisition system design, which considers the integration of sensor, filtering, and information transmission systems, selection of the adequate algorithm, also shows the EMG signals characterization by neural networks and the online and offline prosthetic hand control using such characterizations.

The neural network training is carried out with vectors that contains 20 extracted features from the raw EMG signals, achieving a 95.2% accuracy in practices and validation, and 93% accuracy in a real environment experiment. Although there is a high response reliability percentage and more features were used for training, the response has not been improved significantly compared to other proposals that only use 6 or 7 features in their training vectors. Which leads to question; there exist an optimal evaluating features number to obtain high efficiency without sacrificing time and processing resources?

The system was evaluated with online and offline executions using MATLAB toolbox, in order to have a homogeneous behavior, observing that the offline execution had better execution time, but both of them control adequately the designed prosthesis.

The development of a low-cost hardware system goal is fulfilled, the performance shown is better than some high-cost professional hardware systems. Which is an advantage for prosthesis implementation and encourage their use in poor or developing countries.

Figure 9 shows the entire system developed, i.e., the electronic circuit, the prosthesis and the power supply.

### Future Work

The proposed solution can be extended to explore the sensor channels number, which it may has a direct influence with the extracted features number.

The extracted features number optimization is proposed, and the creation of a prosthesis with materials that not only replicate the hand movements, but also be used by a patient in the future as a real hand, this include a better prosthetic hand development, made with additive manufacturing.

There are different methods to do a system recognition, as shown in [42], that would be worthy to test and compare the performance of those methods versus the proposed in this manuscript.

## Figures and Tables

**Table 1:** EMG signal characteristics, for a hybrid vector of 20 times, frequency, and time-frequency.

20 Characteristics Vector
Mean absolute value
Zero crossing
Slope sign change
Waveform length
Autoregressive model AR (5): first, second, and third coefficients
Fast Fourier Transform: Energy, periodogram, and mean power
Short time Fourier transform, Window 1: Energy, spectrogram, and mean power
Short time Fourier transform, Window 2: Energy, spectrogram, and mean power
Short time Fourier transform, Window 3: Energy, spectrogram, and mean power
Wavelet transform approximation: Coefficient 1 and variance; coefficient 2 and variance; coefficient 3 and variance; coefficient 4 and variance; coefficient 5, variance

**Table 2:** Training characteristics and validation of results to be used in the prosthesis.

TF from MATLAB	Algorithm	Best Performance of Mean Square Error	Epoch of the Best Validated Performance	Accuracy
trainlm	Levenberg-Marquardt	0.010917	12	95.6%
trainbfg	BFGS Quasi-Newton	0.04938	157	78.8%
traincgp	Polak-Ribiére Conjugate Gradient	0.035416	27	88.8%
trainoss	One Step Secant	0.029079	48	91.2%
traingdx	Variable Learning Rate Backpropagation	0.043967	26	90.8%

**Table 3:** Comparison of acquisition systems.

Reference	Acquisition Device	Hand Gestures	Algorithm Characterization	Extracted Features or Training Data	Acquisition Frequency	Success Average Rate
[35]	Biopac MP100 data acquisition system	Left, right, up, down	Back propagation with Levenberg-Marquardt	7 features: MAV, RMS, VAR, SD, ZC, SSC, WT, and db2 with 4 levels	1 kHz	Between 88.4% and 89.2%
[36]	°	Close hand, flex hand, extend the hand and fine grip	Convolutional NN	Images	8 kHz	83.7% ± 13.5%, 71.2% ± 20.2%, 82.6% ± 13.9% and 74.6% ± 15 % for each movement
[37]	MYO armband	Fist, Wave In, Wave Out, Fingers Spread, and Double Tap	feedforward ANN	5 features: MAV, SSC, WL, RMS, and Hjorth parameter (HP)	°	98.7 %
[38]	BIOPAC-MP100C data acquisition system	Left, Right, Up and Down	Levenberg-Marquardt and scaled conjugate gradient based back-propagation	7 features: Moving Average, RMS, VAR, SD, ZC, SSC and WL.	1 kHz	88.4%
[39]	MyoWare Muscle Sensor (AT-04-001)	Cylindrical Grasp, Supination (Twist Left), Pronation (Twist Right), Resting Hand and Open Hand	Back propagation used Lavenberg-Marquardt	4 features: MAV, median, WL and RMS	°	80%
[40]	MyoWare Muscle Sensor	Rock, scissors, paper, one, three, four, good, okay, finger gun, and rest	Multilayer perceptron, support vector machine (SVM), random forest (RF), and a logistic regression (LR).	6 features: RMS, VAR, MAV, SSC, ZC, and WL.	°	Maximum 94%, depending on the methodology
[41]	labVIEW and Biokit	Open and closing, thumb flexion, index flexion, middle and ring finger flexion.	Levenberg-Marquardt	6 features: MAV, RMS, MNF, ZC, SSC and SD.	1 kHz	°
Our project	Own	Closed hand, grip, index, middle, ring.	Back propagation with Lavenberg-Marquardt	20 features: explained above.	1 kHz	95.2% and 93% in real test.

**Table 4:** Identification of 9 random manual gestures executed online and offline comparison.

Movement	Online Execution Time [s]	Offline Execution Time [s]
Trial 1: Index	1.095399	2.352354
Trial 2: Grip	0.874528	2.300595
Trial 3: Closed hand	1.015186	2.208586
Trial 4: Ring finger	1.043519	2.342219
Trial 5: Middle	0.953655	2.816689
Trial 6: Ring finger	1.008239	2.234787
Trial 7: Closed hand	1.069840	2.185594
Trial 8: Grip	1.003771	2.324529
Trial 9: Index	0.893527	2.065108

## Data Availability

Supporting data is available via request to authors.
